# Supplementary material for: Transcription Factors and ncRNAs Associated with CYP3A Expression in Human Liver and Small Intestine Assessed with Weighted Gene Co-Expression Network Analysis
Source: Biomedicines. 2022 Nov 28;10(12):3061. doi: 10.3390/biomedicines10123061 (PMC9775998; doi:10.3390/biomedicines10123061)
Supplement: Supplementary file 1 [file biomedicines-10-03061-s001.zip › Table S4.pdf]

Table S4. Comparison of *CYP3A* associated TFs with or without ncRNAs in liver and small intestine.

CYP3A	Tissue	Group	Number of TFs	Number of overlapping TFs	Number and percentage of different TFs
CYP3A4	Liver	Group 1 with ncRNAs	50	24	26 (52.0%)
		Group 3 without ncRNAs	26		2 (7.7%)
CYP3A5	Liver	Group 1 with ncRNAs	43	24	19 (44.2%)
		Group 3 without ncRNAs	29		5 (17.2%)
CYP3A7	Liver	Group 1 with ncRNAs	45	24	21 (46.7%)
		Group 3 without ncRNAs	27		3 (11.1%)
CYP3A4	Small intestine	Group 2 with ncRNAs	301	270	31 (10.3%)
		Group 4 without ncRNAs	305		35 (11.5%)
CYP3A5	Small intestine	Group 2 with ncRNAs	339	305	34 (10.0%)
		Group 4 without ncRNAs	356		51 (14.3%)
CYP3A7	Small intestine	Group 2 with ncRNAs	22	19	3 (13.6%)
		Group 4 without ncRNAs	31		12 (38.7%)

TFs associated with each *CYP3A* with a weight value greater than 0.05 from group 1 to 4 are obtained. For each *CYP3A*, the numbers of TFs associated with each *CYP3A* are compared with or without ncRNAs in liver and small intestine.
