# Supplementary material for: Transcription Factors and ncRNAs Associated with CYP3A Expression in Human Liver and Small Intestine Assessed with Weighted Gene Co-Expression Network Analysis
Source: Biomedicines. 2022 Nov 28;10(12):3061. doi: 10.3390/biomedicines10123061 (PMC9775998; doi:10.3390/biomedicines10123061)
Supplement: Supplementary file 1 [file biomedicines-10-03061-s001.zip › Table S5.pdf]

Table S5. Comparison of *CYP3A* associated TFs and ncRNAs between three *CYP3As* in liver and small intestine.

Group	*CYP3A*	Number of ncRNAs and TFs	Number of overlapping ncRNAs	Number and percentage of different ncRNAs	Number of overlapping TFs	Number and percentage of different TFs
Group 1 with all genes in liver	*CYP3A4*	66 ncRNAs and 50 TFs	52	14 (21.2%)	43	7 (14.0%)
	CYP3A5	53 ncRNAs and 43 TFs		1 (1.9%)		0 (0.0%)
	CYP3A4	66 ncRNAs and 50 TFs	56	10 (15.2%)	43	7 (14.0%)
	CYP3A7	57 ncRNAs and 45 TFs		1 (1.8%)		2 (4.4%)
	CYP3A5	53 ncRNAs and 43 TFs	51	2 (3.8%)	42	1 (2.3%)
	CYP3A7	57 ncRNAs and 45 TFs		6 (10.5%)		3 (6.7%)
Group 2 with all genes in small intestine	*CYP3A4*	551 ncRNAs and 301 TFs	551	0 (0.0%)	301	0 (0.0%)
	CYP3A5	642 ncRNAs and 339 TFs		91 (14.2%)		38 (11.2%)
	CYP3A4	551 ncRNAs and 301 TFs	0	551 (100%)	0	301 (100%)
	CYP3A7	57 ncRNAs and 22 TFs		57 (100%)		22 (100%)
	CYP3A5	642 ncRNAs and 339 TFs	0	642 (100%)	0	339 (100%)
	CYP3A7	57 ncRNAs and 22 TFs		57 (100%)		22 (100%)

TFs associated with each *CYP3A* with a weight value greater than 0.05 from group 1 (with all genes in liver) to 2 (with all genes in small intestine) are obtained. The numbers of TFs and ncRNAs associated with each *CYP3A* are compared between three *CYP3As* in liver and small intestine. In group 2, *CYP3A4* and *3A5* are in turquoise module and *CYP3A7* in red module, thus no overlapping TFs and ncRNAs are between *CYP3A4* and *3A7* or *CYP3A5* and *3A7*.
