# Supplementary material for: Transcription Factors and ncRNAs Associated with CYP3A Expression in Human Liver and Small Intestine Assessed with Weighted Gene Co-Expression Network Analysis
Source: Biomedicines. 2022 Nov 28;10(12):3061. doi: 10.3390/biomedicines10123061 (PMC9775998; doi:10.3390/biomedicines10123061)
Supplement: Supplementary file 1 [file biomedicines-10-03061-s001.zip › Table S6.pdf]

Table S6. Comparison of *CYP3A* associated ncRNAs and TFs between liver and small intestine.

CYP3A	Tissue	Gene Type	Number of ncRNAs	Number of overlapping ncRNAs	Number and percentage of different ncRNAs	Number of TFs	Number of overlapping TFs	Number and percentage of different TFs
CYP3A4	Liver	All genes	66	15	51 (77.3%)	50	7	43 (86.0%)
	Small intestine	All genes	551		536 (97.3%)	301		294 (97.7%)
CYP3A5	Liver	All genes	53	14	39 (73.6%)	43	8	35 (81.4%)
	Small intestine	All genes	642		628 (97.8%)	339		331 (97.6%)
CYP3A7	Liver	All genes	57	2	55 (96.5%)	45	1	44 (97.8%)
	Small intestine	All genes	57		55 (96.5%)	22		21 (95.5%)

TFs and ncRNAs associated with each *CYP3A* with a weight value greater than 0.05 from group 1 to 2 are obtained. For each *CYP3A*, the numbers of TFs and ncRNAs associated with each *CYP3A* are compared between liver and small intestine.
