# Supplementary material for: Transcription Factors and ncRNAs Associated with CYP3A Expression in Human Liver and Small Intestine Assessed with Weighted Gene Co-Expression Network Analysis
Source: Biomedicines. 2022 Nov 28;10(12):3061. doi: 10.3390/biomedicines10123061 (PMC9775998; doi:10.3390/biomedicines10123061)
Supplement: Supplementary file 1 [file biomedicines-10-03061-s001.zip › Table S7.pdf]

Table S7. Transcriptional factors contributing to *CYP3A* gene expression level in the liver and the small intestine.

			Liver (n = 193)				
Dependent Variable	Independent Variable	Type	Adj. R-Square	*p*-Value	R-Square change	Beta	*p*-Value
CYP3A4			45.07%	7.07×10^{-26}			
	ESR1	humanTF			36.36%	0.400	3.16×10^{-9}
	TEAD2	humanTF			9.28%	-0.366	4.59×10^{-8}
CYP3A5			55.31%	2.24×10^{-31}			
	NR1I2	humanTF			40.48%	0.455	2×10^{-7}
	HLF	humanTF			3.53%	0.430	2.99×10^{-6}
	HMGB3	humanTF			6.44%	-0.409	3.74×10^{-5}
	ARNTL2	humanTF			2.54%	0.461	9.89×10^{-5}
	NFYC	humanTF			2.56%	1.077	5.19×10^{-4}
	PATZ1	humanTF			1.16%	-0.448	2.70×10^{-2}
CYP3A7			25.96%	6.04×10^{-13}			
	CREB3L3	humanTF			3.89%	-0.323	4.33×10^{-3}
	ZBTB47	humanTF			21.53%	-0.854	1.13×10^{-2}
	NR1I3	humanTF			1.70%	0.299	3.71×10^{-2}
			Small Intestine (n = 175)				
Dependent Variable	Independent Variable	Type	Adj. R-Square	*p*-Value	R-Square change	Beta	*p*-Value
CYP3A4			93.82%	9.00×10^{-97}			
	NR1I2	humanTF			3.39%	1.763	3.68×10^{-9}
	FOXA3	humanTF			2.65%	-1.215	1.26×10^{-7}
	ATOH1	humanTF			0.95%	0.755	4.05×10^{-7}
	CREB3L3	humanTF			83.27%	0.406	7.67×10^{-6}
	FOXA1	humanTF			0.34%	-0.878	3.67×10^{-4}
	BARX2	humanTF			0.76%	-0.616	7.63×10^{-4}

	ELF3	humanTF			0.35%	-0.486	9.43×10^{-4}
	ISX	humanTF			0.28%	0.514	5.65×10^{-3}
	ZBTB7B	humanTF			2.16%	0.530	1.31×10^{-2}
CYP3A5			96.01%	1.84×10^{-112}			
	NR1I2	humanTF			92.65%	0.784	1.83×10^{-20}
	FOXA3	humanTF			0.50%	-0.641	1.20×10^{-8}
	MNX1	humanTF			0.45%	0.607	3.28×10^{-6}
	MLXIPL	humanTF			0.92%	0.189	2.94×10^{-5}
	KLF5	humanTF			0.23%	0.263	3.31×10^{-3}
	TOX3	humanTF			0.61%	-0.274	8.02×10^{-3}
	FOXA1	humanTF			0.16%	-0.359	1.11×10^{-2}
	HNF1B	humanTF			0.58%	0.310	1.73×10^{-2}
	ELF3	humanTF			0.12%	0.158	2.18×10^{-2}
CYP3A7			69.32%	1.90×10^{-40}			
	NR6A1	humanTF			56.83%	0.437	1.70×10^{-5}
	RAG1	humanTF			1.35%	0.733	6.07×10^{-5}
	NPAS2	humanTF			2.01%	-0.262	5.50×10^{-4}
	ZNF823	humanTF			1.70%	-0.490	1.37×10^{-3}
	FOXD1	humanTF			0.87%	-0.204	6.52×10^{-3}
	NR3C2	humanTF			2.74%	0.269	8.17×10^{-3}
	ZNF57	humanTF			1.09%	0.317	1.38×10^{-2}
	TULP4	humanTF			4.14%	0.236	3.91×10^{-2}
