# Supplementary figures and images for: Transcription Factors and ncRNAs Associated with CYP3A Expression in Human Liver and Small Intestine Assessed with Weighted Gene Co-Expression Network Analysis
Source: Biomedicines. 2022 Nov 28;10(12):3061. doi: 10.3390/biomedicines10123061 (PMC9775998; doi:10.3390/biomedicines10123061)

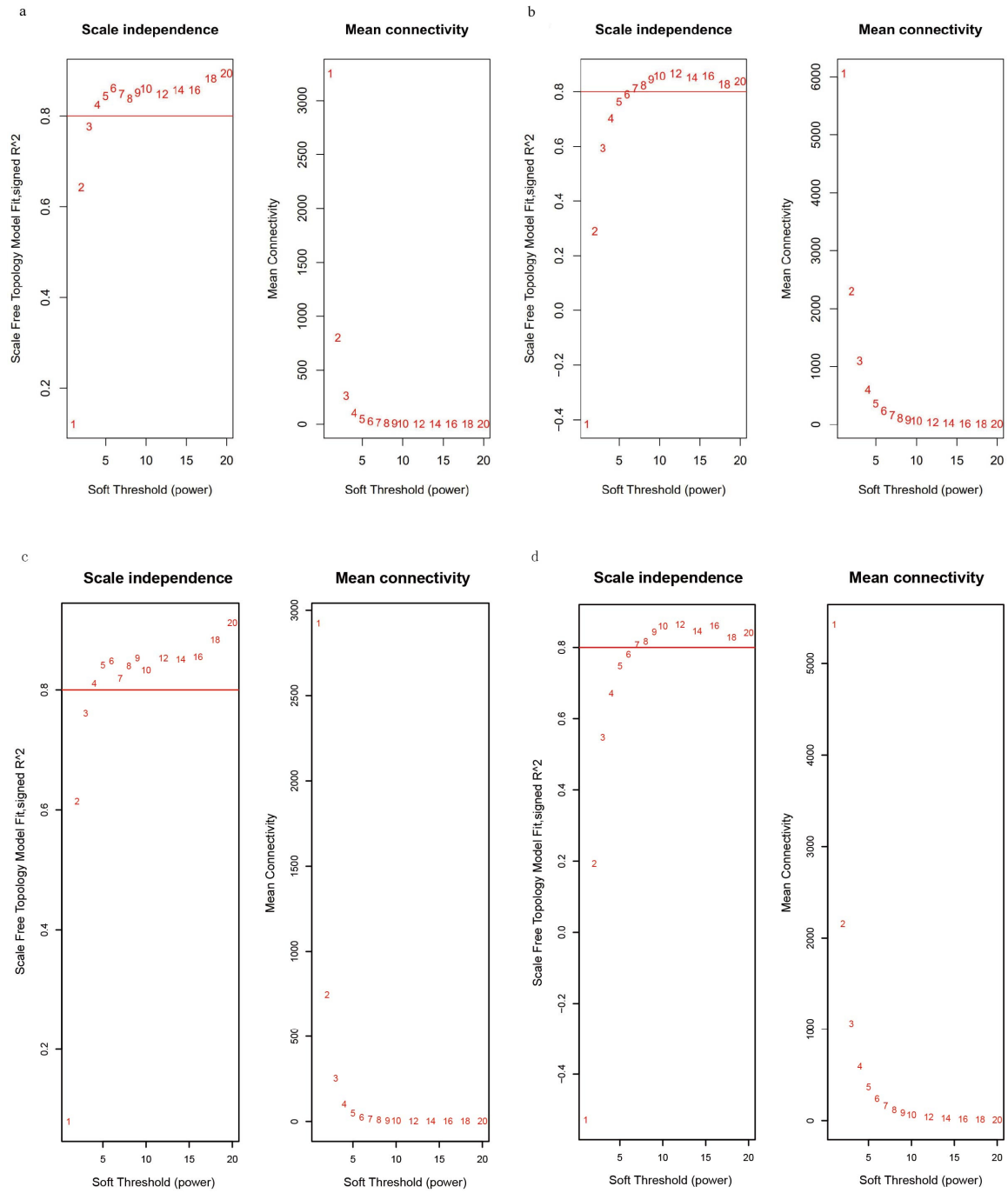

Figure S1. Network topology for different soft-thresholding powers.

Supplement: Supplementary file 1 [file biomedicines-10-03061-s001.zip › Figure S1.pdf]

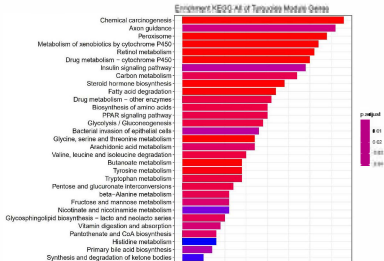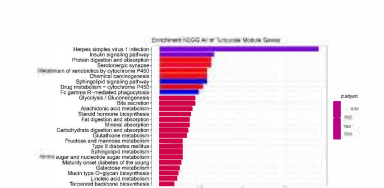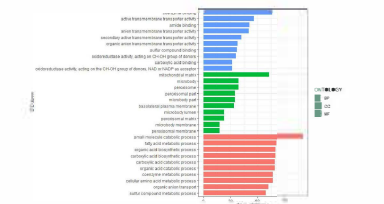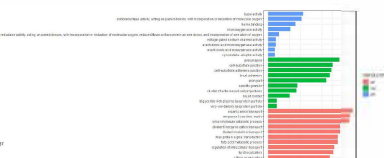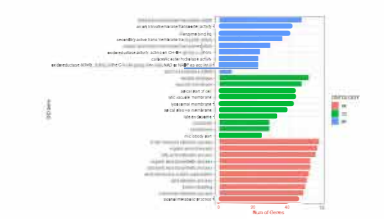

Supplement: Supplementary file 1 [file biomedicines-10-03061-s001.zip › Figure S2.PDF]
